# Artificial Intelligence Outperforms Physicians in General Medical Knowledge, Except in the Paediatrics Domain: A Cross-Sectional Study

**DOI:** 10.3390/bioengineering12060653

**Published:** 2025-06-14

**Authors:** Joana Miranda, Raquel Pereira-Silva, João Guichard, Jorge Meneses, Andreia Neves Carreira, Daniela Seixas

**Affiliations:** Tonic Easy Medical, S.A., 4300-259 Porto, Portugal; joana.miranda@tonicapp.com (J.M.); raquel.silva@tonicapp.com (R.P.-S.); joao.guichard@tonicapp.com (J.G.); andreia.carreira@tonicapp.com (A.N.C.)

**Keywords:** generative artificial intelligence, paediatrics, medical education, large language models, medical knowledge

## Abstract

Generative artificial intelligence (genAI) shows promising results in clinical practice. This study compared a GPT-4-turbo virtual assistant with physicians from Italy, France, Spain, and Portugal on medical knowledge derived from national exams while analysing knowledge retention over time and domain-specific performance. Via a digital platform, 17,144 physicians provided 221,574 answers to 600 exam questions between December 2022 and February 2024. Physicians were stratified by years since graduation and specialty, and the assistant answered the same questions in each native language. Differences in proportions of correct answers were tested with binomial logistic regression (odds ratios, 95% CI) or Fisher’s exact test (α = 0.05). The assistant outperformed physicians in all countries (72–96% vs. 46–62%; logistic regression, *p* < 0.001). Physicians also trailed the assistant across most knowledge domains (*p* < 0.001), except paediatrics (45% vs. 52%; Fisher, *p* = 0.60). Accuracy declined with seniority, falling 4–10% between the youngest and oldest cohorts (logistic regression, *p* < 0.001). Overall, genAI exceeds practising doctors on broad medical knowledge and may help counter knowledge attrition, though paediatrics remains a domain requiring targeted refinement.

## 1. Introduction

In 2022, large language models (LLMs) gained significant attention with the emergence of Chat Generative Pre-Trained Transformer, GPT, which revolutionised conversational artificial intelligence (AI) by retaining the context of interactions. ChatGPT 4’s unprecedented adoption rate—reaching 100 million users within just two months and currently boasting over 130 million users worldwide—highlights its impact [1,2,3].

Physicians were among the many professionals who quickly adopted generative AI (genAI) informally in their practice, an AI field that concentrates on generating new and original information by machine learning on massive databases. GenAI is mainly used as a tool to gather knowledge, provide information, train, or facilitate conversations with patients and their families [4].

A survey conducted by the American Medical Association provided insights into physicians’ engagement with AI tools. The survey revealed high enthusiasm among physicians for tools that reduce administrative burden, with 54% appreciating AI for documentation. Additionally, 41% of physicians expressed a mix of excitement and concern about the potential of the increased use of AI in healthcare [5]. Another study, focused on physician attitudes towards AI in daily practice, revealed that if AI solutions were reliable, fast, and available, 78% of physicians intended to frequently or always use AI for diagnosis, management, or exam interpretation [6]. GenAI is still in its infancy, yet it has been evolving at an unprecedented pace [7].

The applications of genAI in the medical field are extensive, comprising contributions to research, assistance to professionals in clinical and laboratory diagnosis, and updating healthcare practitioners on novel developments in their respective fields. GenAI is also transforming medical education by offering simulation-based learning tools, enabling practitioners to gain hands-on experience in a controlled virtual environment. These innovations are meant to enhance clinical practice, as healthcare systems increasingly integrate genAI to address complex medical challenges [8]. Recent advances have demonstrated that AI can generate medical answers that can match the quality and empathy of responses provided by physicians, with evaluators preferring chatbot responses in nearly 80% of cases [9]. Furthermore, expert-level medical question-answering models such as Med-PaLM 2 have exhibited performance on par with practising clinicians across multiple medical question benchmarks, and their answers were frequently preferred by both physicians and laypeople [10]. These findings underscore a growing recognition that AI, when rigorously evaluated, can meaningfully contribute to medical education, clinical decision support, and patient communication. Nevertheless, there remains a critical need to systematically assess the performance of such AI systems in structured, exam-based scenarios and to benchmark their capabilities against those of experienced clinicians in order to better understand their potential and limitations in real-world medical contexts.

In this study, we aimed to assess the general medical knowledge of physicians from Italy, France, Spain, and Portugal using questions derived from the respective national medical exams and compare their performance with that of a medical AI virtual assistant based on genAI technology. This approach enables a direct, standardised evaluation of AI capabilities relative to experienced clinicians across diverse European healthcare contexts.

## 2. Materials and Methods

To study the general medical knowledge of physicians from Italy, France, Spain, and Portugal and compare their performance with that of an AI virtual assistant, we collected the daily answers from 17,144 physicians to questions taken from national medical exams in each country from December 2022 to February 2024. In this study, there were no missing data.

The questions were published in the medical quiz section of Tonic Easy Medical, a platform and medical device for physicians that curates and aggregates professional resources for their day-to-day work. At the Tonic platform, users must provide accurate and verifiable professional information which, for integrity measures, is subjected to verification. By agreeing with the platform’s privacy policy, users consent that aggregated or de-identified data might be used for research purposes, but no personal identifying information can be processed, respecting the terms of use and privacy policy of the application [11,12] and the EU General Data Protection Regulation (Regulation EU 2016/679). In accordance with this, to provide anonymity, an untraceable random code of identification is given to each user upon registration.

To assess the physicians’ knowledge, we compared the percentage of correct answers achieved by different cohorts across a series of questions from national medical exams. Physicians were categorised into five cohorts, based on years since graduating from medical school: 0 to 10 years; 10 to 20 years; 20 to 30 years; 30 to 40 years; and 40 years or more of experience (Table 1).

The questions were obtained from each country’s national medical exams, except for France, where the exams are not publicly available. The exams Medicina Generale (MMG) and Scienze della Salute e della Medicina (SSM)—Italy, Médico Interno Residente (MIR)—Spain, and Prova Nacional de Acesso (PNA)—Portugal, are designed to evaluate the knowledge of physicians for qualification for specialised medical training. The exams are developed by a group of experts from each country and consist of multiple-choice questions with a single right answer.

For Italy, questions were taken from two exams, the MMG for general practitioners and the SSM for other specialties. In our study, regardless of specialty, physicians answered questions from both the MMG and the SSM. For Spain, questions were sourced from the MIR 2020, 2021, and 2022.

In Portugal, questions were sourced from the PNA 2020, 2021, and 2022. The PNA exam is divided by knowledge domains: internal medicine, surgery, paediatrics, psychiatry, and gynaecology/obstetrics.

French physicians were presented with translated versions of the PNA and SSM exams. The translations of the questions were performed by French, Italian, and Portuguese native physicians to ensure they retained their technical content.

To administer the exam questions to physicians, the selected items were organised by specialty, preserving their original sequence from the national examinations. One question was released per day to all participants on the platform. The release schedule was structured so that questions from different specialties were interleaved and distributed across non-consecutive, randomly assigned days throughout the study period. This approach was designed to minimise potential learning effects and content biases that could arise from sequential or clustered question presentation. Table 2 describes the dataset used in this study.

No questions were excluded from the Portuguese and French exams; all items from these countries were included in the analysis. In Italy, a number of questions were excluded from each exam due to the presence of figures (MMG 2020: 43 questions; MMG 2021: 69; SSM 2021: 45; and SSM 2022: 62), which are not supported by Tonic’s platform. In Spain, a total of 178 questions were excluded across the three MIR exams (67 from MIR 2020, 60 from MIR 2021, and 51 from MIR 2022). Of these, 163 were excluded due to image-based content, while 15 were excluded due to the risk of ambiguous interpretation.

The same set of questions answered by physicians was presented to Tonic’s AI virtual assistant. The AI virtual assistant receives written inputs and provides written outputs. It employs a framework of LLMs to provide the answers. The AI assistant used in this study was based on GPT-4-turbo and operated with a system prompt that instructed it to adopt the persona of a physician, communicating in an informal and collegial manner with other doctors, and to accompany each response with a concise justification that makes its clinical reasoning explicit. These modifications were limited to the assistant’s communication style and operational boundaries, with no alteration to the underlying model or its medical knowledge. No additional fine-tuning or domain-specific training was performed beyond this prompt-based modification. The base GPT-4-turbo model’s parameters remained unchanged, and we did not expose nor train the AI virtual assistant on any of the exam questions used in this evaluation.

**Figure 1:**
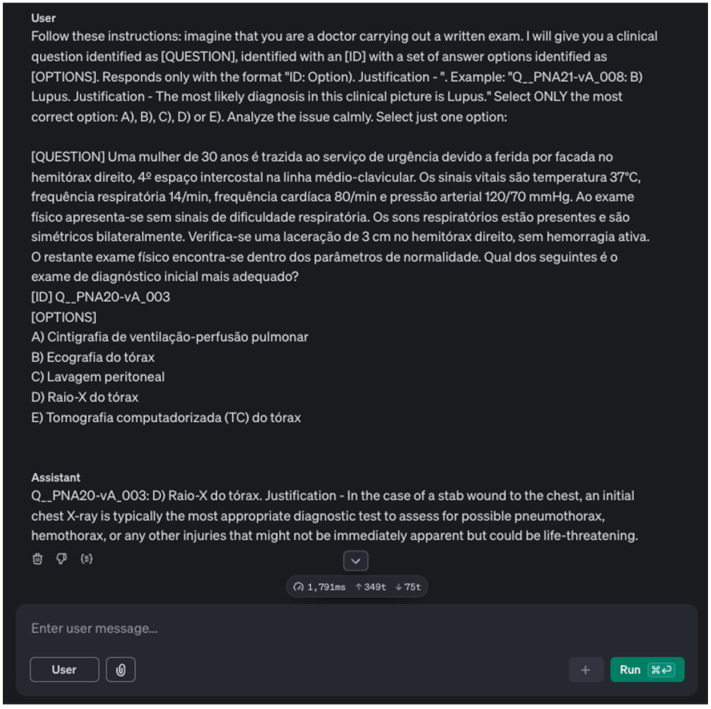
Prompt used for the evaluation of the virtual assistant, illustrating the instructions provided for answering clinical questions, including the expected response format.

To minimise potential translation biases, the questions were provided to the virtual assistant in their native language while using the same prompt, in English, for all exams. The prompt used was as follows: Follow these instructions: Imagine you are a physician performing an exam. I will provide you with a clinical question labelled as [QUESTION], identified with an [ID] with a set of response options identified as [OPTIONS]. Respond with the format “ID: Options. Justification—”. The image illustrating this prompt is shown in Figure 1. This approach enabled us to obtain the answers to all the exams’ questions from the AI virtual assistant and compare them with those provided by the physicians.

To determine if there were significant differences in the distribution of exam scores (ranging from 0 to 100%) across various categorical variables (years since graduation, specialty, country, and knowledge domains (PNA only, internal medicine, surgery, paediatrics, psychiatry, and gynaecology/obstetrics)), we modelled the proportion of correct answers with binomial logistic regression models. Odds ratios with 95% confidence intervals were reported, and statistical significance was set at 0.05. For specific comparisons with small sample sizes or extreme group-size imbalance, namely, the paediatrics domain and the paediatrician-vs.-GP contrast, Fisher’s exact test was used to obtain exact *p*-values. Python 3.12 was used for statistical analysis in this study, utilising the data processing libraries Pandas [13] and NumPy [14]. All statistical data are presented in Supplementary Tables S1–S5.

## 3. Results

From December 2022 to February 2024, 600 exam questions received 221,574 answers from 17,144 physicians. An overview of the physicians’ distribution across countries, years since graduation, and medical specialties is provided in Table 1.

**Figure 2:**
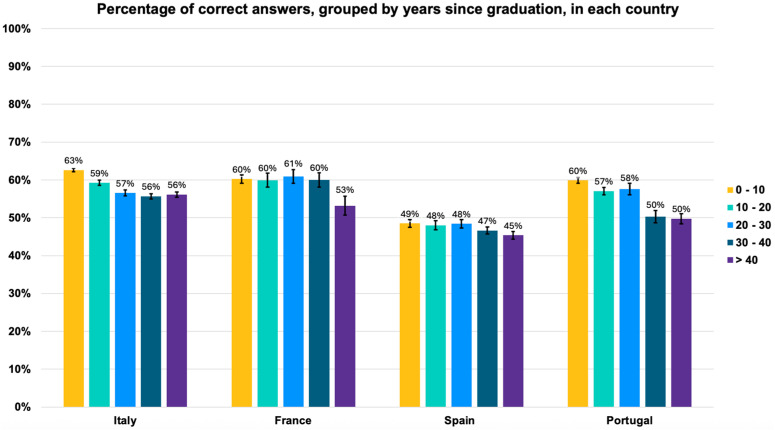
Percentage (%) of correct answers by cohort of physicians based on years since graduation (0–10; 10–20; 20–30; 30–40; ≥40 years). Number of answers by country: 119,868 Italy, 16,877 France, 43,861 Spain, and 40,968 Portugal. Overall accuracy declines progressively with increasing seniority, yielding an odds ratio of 0.93 per decade. Bars represent mean accuracy; error bars indicate 95% confidence intervals. Logistic regression for years since graduation, *p* < 0.001.

Accessing the number of correct answers, there was a statistically significant decline in knowledge across physicians of all countries with increasing years since graduation (Figure 2). In Spain, this decline was of 4% (from 49% [95% CI: 48.0–49.7] in the 0–10-year cohort to 45% [95% CI: 44.0–46.0] in the ≥40-year cohort). In Italy and France, the decline was 7% (in Italy from 63% [95% CI: 62.6–63.4] to 56% [95% CI: 55.3–56.7] and in France from 60% [95% CI: 58.9–61.1] to 53% [95% CI: 50.4–55.5]). In Portugal, the decline in general medical knowledge was the most pronounced, reaching 10%, from 60% [95% CI: 59.3–60.7] to 50% [95% CI: 48.6–51.3]).

To formally assess these effects, we performed a multivariable logistic regression on the cohort-level counts, using correct answer as the binary outcome and adjusting for country and years since graduation. Each additional decade since graduation was associated with a 7% decrease in the odds of a correct answer (β = –0.0072; adjusted OR per decade = 0.93, 95% CI 0.92–0.94; *p* < 0.001; Supplementary Table S1).

Since physicians in France were exposed to the exams from Portugal and Italy, a direct comparison between the performance of physicians by country was possible. In the Portuguese exam PNA 2021, French physicians performed better than the Portuguese physicians. French physicians answered correctly 62% of the questions (95% CI: 60.5–63.5), while Portuguese physicians answered correctly 55% of the questions (95% CI: 54.2–55.8) (logistic regression, *p* < 0.001; adjusted OR = 1.34, 95% CI 1.23–1.46; Supplementary Table S2).

**Figure 3:**
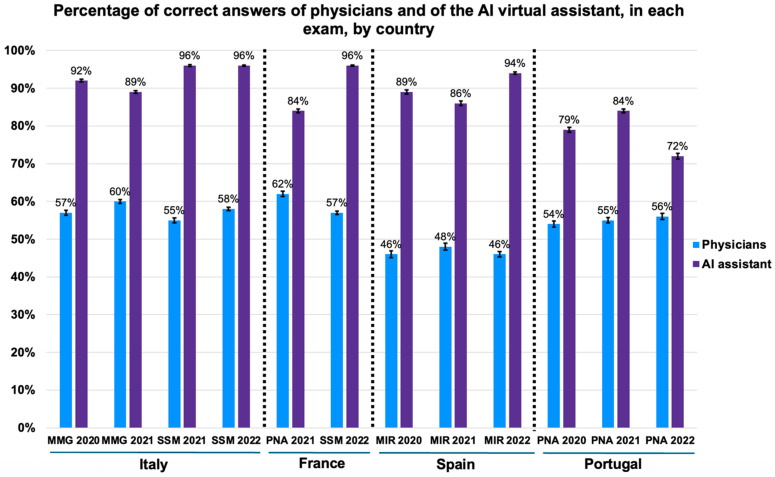
Comparison of the percentage (%) of correct answers of physicians (blue) and of the AI virtual assistant (purple) by exam. Number of answers by medical exam: 12,772 MIR 2020, 12,265 MIR 2021, 18,824 MIR 2022, 22,756 MMG 2020, 36,576 MMG 2021, 21,530 SSM 2021, 49,332 SSM 2022, 14,349 PNA 2020, 19,314 PNA 2021, and 13,856 PNA 2022. In France, physicians responded to PNA 2021 and SSM 2022. Across all twelve exams, the AI assistant achieved higher accuracy than physicians. Bars represent mean accuracy; error bars indicate 95% confidence intervals. Logistic regression adjusted for country and exam, *p* < 0.001. MIR: Médico Interno Residente; MMG: Medicina Generale; PNA: Prova Nacional de Acesso; SSM: Scienze della Salute e della Medicina.

Comparing the performance of physicians with that of the AI virtual assistant, the latter consistently outperformed physicians in all twelve medical exams, obtaining more correct answers (logistic regression, *p* < 0.001; adjusted OR = 7.46, 95% CI 6.43–8.66; Supplementary Table S2), as illustrated in Figure 3. The AI’s accuracy varied from 72% to 96% (95% CI range: 56.2–99.4), while the physicians’ accuracy was between 46% and 62% (95% CI range: 45.1–63.5). Remarkably, in SSM 2021 (distributed to Italian physicians) and SSM 2022 (distributed to Italian and French physicians), the AI had over 95% of the answers correct (SSM 2021: 95%, 95% CI: 82.8–99.4; SSM 2022: 96%, 95% CI: 86.5–98.9).

**Figure 4:**
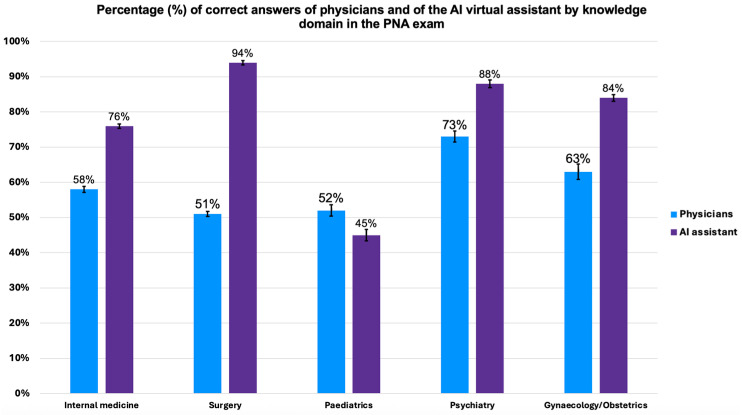
Comparison of the percentage (%) of correct answers of physicians (blue) and of the AI virtual assistant (purple) by knowledge domain in the PNA exam. The questions of the PNA exam are divided by area: internal medicine, surgery, paediatrics, psychiatry, and gynaecology/obstetrics. The number of answers to the PNA in Portugal was 40,968: 19,111 internal medicine, 2002 surgery, 3706 paediatrics, 3265 psychiatry, and 12,884 gynaecology/obstetrics. The analysis was not performed for the French physicians, who also were exposed to the same exam, because the sample was too small. The AI assistant outperformed physicians in four of the five domains; paediatrics showed no difference. Bars represent mean accuracy; error bars indicate 95% confidence intervals. Logistic regression for the domain main effect, *p* < 0.001; paediatrics comparison (Fisher exact), *p* = 0.60. PNA: Prova Nacional de Acesso.

This study further compared the performance of physicians and the AI virtual assistant across knowledge domains. This was possible because the Portuguese national exam (PNA) is divided by knowledge domain: internal medicine, surgery, gynaecology/obstetrics, psychiatry, and paediatrics. The analysis was only conducted for the Portuguese physicians and not for French physicians (who were exposed to Portuguese and Italian exams) due to the small sample size of the latter (43 questions across all domains). The overall performance comparison is illustrated in Figure 4.

The AI virtual assistant demonstrated superior performance compared to physicians in all domains, with a statistically significant difference (logistic regression, *p* < 0.001; Supplementary Table S3), except for paediatrics. In internal medicine, the AI virtual assistant answered 76% of the questions correctly (95% CI: 64.4–85.0), while the physicians answered 58% (95% CI: 57.3–58.7). In surgery, the AI virtual assistant had a 94% accuracy rate (95% CI: 84.8–97.5), much higher than the 51% of the physicians (95% CI: 48.8–53.2). In psychiatry, the AI virtual assistant achieved 88% accuracy (95% CI: 76.9–93.4), compared to 73% of the physicians (95% CI: 71.4–74.5). In gynaecology/obstetrics, the AI virtual assistant reached 84% accuracy (95% CI: 73.2–91.1), while the physicians scored 63% (95% CI: 62.2–63.8).

In contrast, although not reaching statistical significance (Fisher exact test, *p* = 0.60), physicians in paediatrics tended to outperform the AI virtual assistant, with 52% (95% CI: 50.4–53.6) and 45% (95% CI: 32.8–56.7) correct answers, respectively. We further analysed the performance of physicians who treat the paediatric population in the paediatrics domain. Although not statistically significant (Fisher exact test, *p* = 0.56; Supplementary Table S4), paediatricians slightly outperformed general practitioners (GPs) by answering 52% of the questions correctly (95% CI: 43.0–61.4) compared to 49% by the GPs (95% CI: 46.1–52.0).

To deepen the domain analysis, we computed a domain-adjusted logistic regression model comparing AI and physician performance across the five PNA areas (Supplementary Table S3). The overall adjusted OR for AI versus physicians was 2.88 (95% CI 2.39–3.46; *p* < 0.001), whereas the paediatrics-specific OR was 0.80 (95% CI 0.40–1.59), confirming the absence of a significant difference in that domain.

## 4. Discussion

Our study revealed that the AI virtual assistant consistently outperformed physicians in general medical knowledge across most domains in four countries, whereas this pattern did not extend to paediatrics. In this domain, physicians answered a higher percentage of questions correctly than the AI, but this difference was not statistically significant. Additionally, we observed a statistically significant decline in knowledge among physicians across all countries with increasing years since graduation, with knowledge declining by 4% in Spain, 7% in Italy and France, and 10% in Portugal. Our study is based on a large sample size that covers all medical specialties from several countries and includes physicians with a wide range of years of experience.

### 4.1. Medical Knowledge Decline

Our study revealed a significant decline in general medical knowledge among physicians as the years since graduation increased. Interestingly, in another study, Zupanic et al. evaluated the knowledge retention of German GPs based on time elapsed since graduation using the Progress Test Medicine, a systematic feedback instrument that regularly tests medical students with graduate-level questions. This study analysed the performance of 161 GPs on 200 standardised test questions, concluding that recent graduates had better performances. Additionally, it noted that a GP’s knowledge level is moderately influenced by exam grades, time since graduation, the institutional affiliation of postgraduate training, and medical specialisation [15]. This highlights the importance of ongoing education and the potential role of AI in providing up-to-date knowledge support for practising physicians.

To the best of our knowledge, our study is the first large-scale, multi-country evidence that knowledge attrition among physicians is not isolated but a persistent challenge across European healthcare. Our data underscore the urgent need for innovative strategies, such as AI-powered tools, to support lifelong learning and maintain clinical standards. Finally, in this study, we performed multivariable logistic regression to analyse the decline in physician performance over time, adjusting for country and years since graduation. While physician specialty was collected, the sample sizes within individual specialty-by-cohort subgroups were too small to support further stratified or adjusted analyses involving specialty. Additionally, clinical workload data (e.g., patient volume or working hours) were not available. Both factors may influence medical knowledge retention and represent important directions for future research.

### 4.2. Reliability of AI-Based Tools Beyond ChatGPT

In our analysis, AI outperformed physicians in general medical knowledge, setting a benchmark by demonstrating that AI’s superior performance is robust across national contexts, specialties, and experience levels. This breadth and consistency have not been reported before, establishing the generalisability of AI’s knowledge advantage in real-world settings. Nonetheless, other recent studies, such as Bommineni et al., evaluated the performance of ChatGPT on the Medical College Admissions Test (MCAT), an exam for medical students in the United States, Australia, Canada, and the Caribbean Islands, and revealed that ChatGPT performs at or above the median performance of the MCAT physician takers [16]. Another study evaluated the performance of ChatGPT on the United States Medical Licensing Exam, concluding that ChatGPT performed at or near the passing threshold of 60% accuracy without specialised input from human trainers. Furthermore, ChatGPT displayed comprehensible reasoning and valid clinical insights, suggesting that LLMs may potentially assist in medical education settings as a prelude to future integration into clinical decision-making [17]. Other genAI models have also demonstrated positive results in medical knowledge assessments; for instance, Bard answered an average of 62.4% of questions correctly on the ophthalmology board certification exam, although it still lacks the holistic understanding of a trained ophthalmologist [18]. In our study, we used exclusively ChatGPT; however, given the variety of conversational AI systems available, it is also crucial to analyse and compare their performance. Farhat et al. compared Bard, ChatGPT-3.5, and GPT-4 on the National Eligibility cum Entrance Test (NEET), a key Indian medical entrance exam. Notably, GPT-4 passed the NEET-2023 with the highest accuracy. In contrast, ChatGPT-3.5 and Bard showed variations in their performances, with specific strengths in certain subjects and topics [18].

### 4.3. Exploring the Case of Paediatrics

In the present study, despite its impressive overall performance, genAI failed to outperform physicians in paediatrics. Indeed, data show that physicians achieved a higher percentage of correct answers compared to AI, though this difference was not statistically significant. Although this trend was not conclusive, it may indicate an area where AI performance remains limited, suggesting the need for further domain-specific evaluation before clinical deployment in paediatrics. Further investigation is needed to determine the putative limitations of genAI in the specific paediatrics domain. Although the performance difference between AI and physicians in this domain was not statistically significant, we conducted a qualitative review of the seven paediatrics questions answered incorrectly by the AI to explore potential areas for improvement, which revealed recurring issues in the interpretation of age-specific presentations, diagnostic prioritisation, and clinical reasoning that appeared to reflect adult-focused patterns. While these findings are exploratory, they suggest that the paediatrics domain may indeed require additional model adaptation. Curiously, recent studies seem to be in line with our results. Barile et al. examined the diagnostic accuracy of ChatGPT-3.5 in 100 paediatric medical cases and showed that while ChatGPT could identify relevant organ systems, it misdiagnosed 83% of cases. The authors suggested that while the current performance is inadequate for clinical use, further training on the reliable medical literature and having real-time access to accurate data could enhance its clinical utility [19]. Rader et al. revisited this publication, leveraging GPT-4’s enhanced complex reasoning capabilities. While replicating this study with its original cases, GPT-4 achieved a diagnostic accuracy of 37%, more than doubling GPT-3.5’s performance [20]. Another study compared the performance of GPT-3.5 and GPT-4 to that of 849 physicians in the 2022 Israeli board residency examinations across five core medical disciplines: internal medicine, general surgery, paediatrics, psychiatry, and gynaecology/obstetrics. GPT-4 outperformed most physicians in psychiatry and achieved comparable results to the median physician in general surgery and internal medicine. However, its performance was lower in paediatrics and gynaecology/obstetrics, with median percentiles of 17.4% and 23.44%, respectively [21]. Interestingly, despite our study employing a different method to compare results, the AI virtual assistant performed more similarly to paediatrics than the aforementioned study, which may be attributed to the use of the more advanced GPT-4 Turbo model. Nevertheless, the trend that paediatricians outperform AI remains consistent across studies [21], highlighting the need for further refinement of AI in medicine. The nuanced and highly specific nature of paediatrics, along with the individual variability of patients, presents unique challenges that AI has yet to learn to overcome.

Several factors may contribute to the AI’s relatively lower performance in paediatrics. First, AI models often face challenges due to limited and less diverse paediatric datasets, which can restrict their diagnostic accuracy compared to adult medicine [22]. Second, paediatric clinical scenarios require nuanced understanding of developmental physiology and age-specific presentations, which may not be fully captured by general AI training [23]. Finally, generative AI models show promise in paediatric triage and diagnosis but require fine-tuning and domain adaptation to reach optimal reliability [24]. These factors together likely contribute to the relative AI underperformance in this specialty, warranting further research and tailored model development. While our findings do not demonstrate a statistically significant difference, the observed trend aligns with previous studies and underscores the importance of developing and validating AI systems specifically for paediatric medicine.

### 4.4. On ChatGPT Fine-Tuning and the Potential of Applied Prompt Engineering

In our study, we assessed the performance of the medical AI virtual assistant system without the use of prompt engineering or model fine-tuning techniques. The results presented here reflect the AI’s baseline capabilities as deployed and do not provide empirical evidence regarding the impact of prompt engineering or customisation. Recent research, however, has demonstrated that prompt engineering can be an effective strategy to enhance the performance of LLMs in medical question-answering tasks. For instance, Nori et al. systematically evaluated advanced prompting techniques and showed that GPT-4, when guided by the Medprompt approach, outperformed specialist-tuned models such as Med-PaLM 2 on medical benchmarks, while also achieving greater computational efficiency. These findings indicate that prompt design is a practical tool for further improving clinical AI systems [25]. Although our study did not empirically assess prompt engineering or fine-tuning, the evidence from the literature suggests that future iterations of our system could benefit from such approaches. Specifically, leveraging domain-specific knowledge and optimised prompts may lead to additional performance gains, particularly in specialised or challenging domains. Evaluating these strategies within our system remains an important direction for future research.

While AI may excel in general medical knowledge, its performance in specific fields and with individual patients is more challenging, revealing the need for significant and continuous improvement and training of models for healthcare applications. Moreover, the ongoing embedding of comprehensive and highly specialised databases, coupled with the certification as a medical device, builds a safe, reliable, and robust tool to be used by physicians in real clinical settings. Nonetheless, the finding that AI outperformed physicians in most domains suggests a potential role for AI-based tools in medical education and continuing professional development. AI could be used to supplement traditional training methods by offering personalised feedback and up-to-date knowledge support. Integrating AI into educational programs may help address knowledge gaps and support lifelong learning for medical professionals. Furthermore, in addition to the educational element, other AI advances in ensemble deep learning and hybrid AI models show that AI integration in clinical workflows can also enhance diagnostic accuracy and clinical decision-making [26,27]. Indeed, in a study for COVID-19 diagnosis, stacked ensemble models integrating chest X-ray analysis with symptom data show 95–99% accuracy in detecting pulmonary manifestations, surpassing both individual deep learning models and clinician assessments [27]. By rigorously benchmarking AI and physician performance across multiple countries and specialties, our study provides a foundation for future research, regulatory standards, and targeted AI development. These results position our study as a reference point for safe and effective AI integration into clinical practice and medical education.

### 4.5. Known Limitations of This Study

There are some limitations to our study that should be acknowledged. First, in our study, physicians were not in a controlled examination setting, answering a continuous sequence of questions in an exam that significantly impacts their professional careers. Instead, physicians were presented with one question per day and were responding through a mobile app, which could have reduced the accuracy of human responses. This absence of a controlled testing environment for physician participants is relevant inasmuch as factors such as differences in testing conditions, distractions, or access to resources may have contributed to increased response variability among physicians. This variability was not statistically adjusted for in our analysis and should be considered when interpreting the comparative results between physicians and the AI system.

A proportion of questions from the Italian and Spanish exams was excluded due to platform limitations. In Italy, exclusions were due to unsupported image-based content on the Tonic platform. In Spain, most exclusions were also related to image-based questions, with a small number excluded for ambiguous interpretation, which were deemed questionable by Spanish physicians, both in discussion forums and the ones working with us. These accounted for approximately 18% of Italian and 22% of Spanish questions, while all Portuguese and French questions were included. The excluded questions were distributed across domains and not concentrated in any specialty, minimising the risk of systematic bias. However, these exclusions should be considered when interpreting performance comparisons for Italy and Spain.

Regarding sample randomisation, although questions were presented according to their sequence in the original exams, the delivery schedule involved interleaving questions from different specialties and distributing them on randomly assigned, non-consecutive days, which intended to minimise learning effects and content biases that could arise from sequential or clustered question presentation. Nonetheless, as full randomisation of question order was not implemented, some residual risk of order effects cannot be entirely excluded. Future studies may benefit from employing complete randomisation to further address this methodological consideration.

It is important to note that our study focused on real questions from general medical knowledge exams and included physicians with diverse levels of seniority. Indeed, if data from actual exams had been used, it would have limited sample diversity, as it would have only included newly graduated physicians. Nonetheless, although our sample included physicians with a range of years since graduation, we observed a notable concentration of participants with 0–10 years of experience in most countries. This pattern likely reflects our recruitment strategy, which relied on voluntary participation through a mobile app platform and may have attracted younger, more technologically engaged physicians. As a result, there are some limitations regarding the representativeness of our sample with respect to the broader physician population. Interestingly, Spain differed from this trend, displaying a more balanced distribution across experience cohorts and representing a good internal control in this study.

A key limitation of research with LLMs such as GPT-4-turbo is the opacity of their pretraining data. Although we did not perform any additional training of the model for this study, and the AI assistant’s responses relied solely on prompt-based adjustments to communication style, we cannot independently verify or control the content included in the original training corpus. It is therefore possible that some public medical exam questions used in our evaluation were accessible online prior to the model’s knowledge cutoff and could have been present in its pretraining data. This inherent limitation of closed-source AI models should be considered when interpreting our findings.

Another important limitation of current LLMs in clinical applications is their underperformance in fields with pronounced patient variability, such as paediatrics. This can be attributed to several factors. First, paediatric medicine encompasses a wide spectrum of physiological stages and developmental milestones, leading to highly variable disease presentations and management strategies. Generalist AI models are often trained predominantly on adult-centric data, resulting in less exposure to the unique clinical scenarios encountered in paediatric care. Second, the subtleties of age-specific symptoms and the need for tailored diagnostic reasoning may not be sufficiently captured in the training data or model architecture. Third, paediatric datasets are often smaller and less diverse due to privacy concerns and lower prevalence of certain conditions, further limiting the model’s ability to generalise. These challenges underscore the importance of developing domain-specific datasets and fine-tuning strategies to enhance AI performance in paediatrics and other fields characterised by nuanced patient variability.

Furthermore, LLMs pose significant ethical challenges in medical applications, including biased training data, lack of transparency, and potential misdiagnosis. The integration of AI in medical practice must adhere to ethical standards and regulatory frameworks, such as the European AI Act, ensuring transparency, accountability, and patient data protection. We endorse the European Union’s efforts in regulating AI through the European AI Act, which is crucial for ensuring ethical, safe, and rights-respecting AI development and deployment. Throughout this study, compliance with ethical considerations and regulatory requirements was a primary focus and will remain essential in future AI system development [28].

## 5. Conclusions

This study showed that genAI outperformed physicians from four different countries in accurately answering national medical exam questions. Notably, we observed a decline in general medical knowledge among physicians as the years since graduation increased. These findings highlight the potential of genAI as a supportive tool for medical practice, enhancing diagnostic accuracy and decision-making efficiency and saving time. It is essential to emphasise that genAI virtual assistants are designed to collaborate with physicians, augmenting their capabilities rather than replacing them.

Despite AI’s overall strong performance, physicians were not outperformed in paediatrics. Although the difference was not statistically significant, this finding highlights an area where further improvement and targeted development of AI models are needed to ensure safe and effective application in paediatric care. Moreover, ensuring regulatory compliance will be crucial for the safe and effective deployment of AI in healthcare settings.

## Figures and Tables

**Table 1:** Distribution of the number and percentage (%) of medical professionals by country, years since graduation, and specialty. Physicians without specialty are physicians who registered at the platform and labelled themselves as not having a formal medical specialisation.

	Total	Italy	France	Spain	Portugal
Number of physicians	17,144	8427	1468	3594	3655
Distribution of physicians in years since graduation					
0–10 years	41%	46%	44%	23%	45%
10–20 years	15%	11%	16%	15%	24%
20–30 years	12%	10%	17%	17%	10%
30–40 years	17%	17%	15%	24%	9%
+40 years	16%	16%	9%	22%	13%
Distribution of physicians by specialty					
Family medicine	28%	18%	46%	40%	30%
Physicians without specialty	20%	21%	4%	13%	31%
Internal medicine	5%	5%	1%	5%	7%
Anaesthesiology	4%	5%	3%	2%	2%
Paediatrics	3%	3%	2%	3%	3%
General surgery	3%	3%	1%	2%	2%
Gynaecology/obstetrics	2%	2%	1%	2%	2%
Psychiatry	2%	2%	4%	2%	1%
Cardiology	2%	3%	1%	1%	1%
Other specialties	31%	38%	37%	30%	21%

**Table 2:** Distribution of the number of questions and answers by exam and country.

	Questions	Answers
Italy	159	119,868
MMG 2020	30	22,756
MMG 2021	50	36,576
SSM 2021	29	21,530
SSM 2022	50	39,006
France	108	16,877
PNA 2021	43	6551
SSM 2022	65	10,326
Spain	140	43,861
MIR 2020	39	12,772
MIR 2021	39	12,265
MIR 2022	62	18,824
Portugal	193	40,968
PNA 2020	63	14,349
PNA 2021	60	12,763
PNA 2022	70	13,856

Legend: MIR: Médico Interno Residente; MMG: Medicina Generale; PNA: Prova Nacional de Acesso; SSM: Scienze della Salute e della Medicina.

## Data Availability

Individual participant data will not be available since no personal data were processed as part of this investigation. However, other documents, such as the study protocol and statistical analysis, will be accessible. These documents will be available immediately following publication, with no end date for access. The data will be available to anyone who wishes to access it for any type of analysis. Requests for access should be directed to science@tonicapp.com.

## Supplementary Materials

The following supporting information can be downloaded at: https://www.mdpi.com/article/10.3390/bioengineering12060653/s1, Table S1: Retention of knowledge since graduation; Table S2: Overall accuracy of Artificial intelligence (AI) vs physicians in national exams; Table S3: Portuguese PNA: AI vs physicians across knowledge domains; Table S4: Physician specialty vs paediatrics; Table S5: Sensitivity analysis: exam-level aggregation.

## Abbreviations

The following abbreviations are used in this manuscript:

AI	Artificial intelligence.
GenAI	Generative artificial intelligence.
GPT	Generative pre-trained transformer.
LLMs	Large language models.
PNA	Prova Nacional de Acesso (Portuguese national medical exam).
MIR	Médico Interno Residente (Spanish national medical exam).
MMG	Medicina Generale (Italian general medicine exam).
SSM	Scienze della Salute e della Medicina (Italian health and medicine exam).

## References

[1] OpenAI (2023). GPT-4 OpenAI. https://openai.com/index/gpt-4-research/.

[2] Similarweb (2024). Chat.Openai.Com Traffic Analytics, Ranking & Audience. Similarweb.

[3] DeVon C. On ChatGPT’s One-Year Anniversary, It Has More Than 1.7 Billion Users—Here’s What It May Do Next. https://www.cnbc.com/2023/11/30/chatgpts-one-year-anniversary-how-the-viral-ai-chatbot-has-changed.html.

[4] Yim D., Khuntia J., Parameswaran V., Meyers A. (2024). Preliminary Evidence of the Use of Generative AI in Health Care Clinical Services: Systematic Narrative Review. JMIR Med. Inform..

[5] American Medical Association, American Medical Association (2023). In Press: Physicians Enthusiastic But Cautious About Health Care AI.

[6] Giavina-Bianchi M., Amaro E., Machado B.S. (2024). Medical Expectations of Physicians on AI Solutions in Daily Practice: Cross-Sectional Survey Study. JMIRx Med.

[7] Aydın Ö., Karaarslan E. (2023). Is ChatGPT Leading Generative AI? What is Beyond Expectations?. Acad. Platf. J. Eng. Smart Syst..

[8] Dave T., Athaluri S.A., Singh S. (2023). ChatGPT in medicine: An overview of its applications, advantages, limitations, future prospects, and ethical considerations. Front. Artif. Intell..

[9] Ayers J.W., Poliak A., Dredze M., Leas E.C., Zhu Z., Kelley J.B., Faix D.J., Goodman A.M., Longhurst C.A., Hogarth M. (2023). Comparing Physician and Artificial Intelligence Chatbot Responses to Patient Questions Posted to a Public Social Media Forum. JAMA Intern. Med..

[10] Singhal K., Tu T., Gottweis J., Sayres R., Wulczyn E., Amin M., Hou L., Clark K., Pfohl S.R., Cole-Lewis H. (2025). Toward expert-level medical question answering with large language models. Nat. Med..

[11] Medical T.E. Privacy Policy. https://tonicapp.io/privacy-policy/.

[12] Medical T.E. Terms of Use. https://tonicapp.io/terms-of-use/.

[13] The Pandas Development Team (2024). Pandas—Python Data Analysis, Version v2.3.0.

[14] Harris C.R., Millman K.J., van der Walt S.J., Gommers R., Virtanen P., Cournapeau D., Wieser E., Taylor J., Berg S., Smith N.J. (2020). Array programming with NumPy. Nature.

[15] Zupanic M., Kreuer J., Bauer D., Nouns Z.M., Ehlers J.P., Fischer M.R. (2020). Spontaneously retrievable knowledge of German general practitioners depending on time since graduation, measured with the progress test medicine. GMS J. Med. Educ..

[16] Bommineni V.L., Bhagwagar S., Balcarcel D., Bommineni V., Davazitkos C., Boyer D. (2023). Performance of ChatGPT on the MCAT: The Road to Personalized and Equitable Premedical Learning. MedRxiv.

[17] Kung T.H., Cheatham M., Medenilla A., Sillos C., De Leon L., Elepano C., Madriaga M., Aggabao R., Diaz-Candido G., Maningo J. (2023). Performance of ChatGPT on USMLE: Potential for AI-assisted medical education using large language models. PLoS Digit. Health.

[18] Farhat F., Chaudhry B.M., Nadeem M., Sohail S.S., Madsen D.O. (2024). Evaluating Large Language Models for the National Premedical Exam in India: Comparative Analysis of GPT-3.5, GPT-4, and Bard. JMIR Med. Educ..

[19] Barile J., Margolis A., Cason G., Kim R., Kalash S., Tchaconas A., Milanaik R. (2024). Diagnostic Accuracy of a Large Language Model in Pediatric Case Studies. JAMA Pediatr..

[20] Rader B., Hswen Y., Brownstein J.S. (2024). Further Reflections on the Use of Large Language Models in Pediatrics. JAMA Pediatr..

[21] Katz U., Cohen E., Shachar E., Somer J., Fink A., Morse E., Shreiber B., Wolf I. (2024). GPT versus Resident Physicians—A Benchmark Based on Official Board Scores. Nejm Ai.

[22] Kandaswamy S., Knake L.A., Dziorny A., Hernandez S., McCoy A.B., Hess L.M., Orenstein E., White M.S., Kirkendall E.S., Molloy M. (2025). Pediatric Predictive Artificial Intelligence Implemented in Clinical Practice from 2010–2021: A Systematic Review. Appl. Clin. Inform..

[23] Ramgopal S., Sanchez-Pinto L.N., Horvat C.M., Carroll M.S., Luo Y., Florin T.A. (2023). Artificial intelligence-based clinical decision support in pediatrics. Pediatr. Res..

[24] Ho B., Lu M., Wang X., Butler R., Park J., Ren D. (2025). Evaluation of Generative Artificial Intelligence Models in Predicting Pediatric Emergency Severity Index Levels. Pediatr. Emerg. Care.

[25] Nori H., Lee Y.T., Zhang S., Carignan D., Edgar R., Fusi N., King N., Larson J., Li Y., Liu W. (2023). Can Generalist Foundation Models Outcompete Special-Purpose Tuning? Case Study in Medicine. arXiv.

[26] Bozyel S., Simsek E., Kocyigit Burunkaya D., Guler A., Korkmaz Y., Seker M., Erturk M., Keser N. (2024). Artificial Intelligence-Based Clinical Decision Support Systems in Cardiovascular Diseases. Anatol. J. Cardiol..

[27] AlMohimeed A., Saleh H., El-Rashidy N., Saad R.M.A., El-Sappagh S., Mostafa S. (2023). Diagnosis of COVID-19 Using Chest X-ray Images and Disease Symptoms Based on Stacking Ensemble Deep Learning. Diagnostics.

[28] The European Parliament, The European Parliament (2024). Artificial intelligence act. Regulation (EU) 2024/1689.

