# Supplementary material for: Artificial Intelligence Outperforms Physicians in General Medical Knowledge, Except in the Paediatrics Domain: A Cross-Sectional Study
Source: Bioengineering (Basel). 2025 Jun 14;12(6):653. doi: 10.3390/bioengineering12060653 (PMC12190018; doi:10.3390/bioengineering12060653)
Supplement: Supplementary file 1 [file bioengineering-12-00653-s001.zip › bioengineering-3579907-supplementary.pdf]

Supplementary Materials:

Supplementary Table S1. Retention of knowledge since graduation

Predictor	β (SE)	Adjusted OR	95 % CI	*p*
Years since graduation (per 10 years)	–0.072 (0.003)	0.93	0.92 – 0.94	<0.001
Country fixed effects	—	—	—	all < 0.01
Model N (physician answers)	221 574			

Logistic regression of physician answers, with correct vs incorrect *as the outcome, weighted by the number of answers in each years-since-graduation × country group. The key predictor is years since graduation (per 10-year increase). β – regression coefficient; CI – confidence interval; OR – odds ratio; SE – standard error.*

Supplementary Table S2. Overall accuracy of Artificial intelligence (AI) vs physicians in national exams

Predictor	β (SE)	Adjusted OR	95 % CI	*p*
Responder = AI (reference)	—	—	—	—
Physician	–2.01 (0.08)	0.13	0.12 – 0.16	<0.001
Country = Italy (reference)	—	—	—	—
France	–0.07 (0.02)	0.93	0.89 – 0.97	0.001
Spain	–0.30 (0.02)	0.74	0.71 – 0.77	<0.001
Portugal	–0.05 (0.02)	0.95	0.92 – 0.99	0.013
Exam indicators (11 dummies)	—	—	—	all < 0.05
Model N (answers)	228 774			

Logistic regression with correct vs incorrect *as the outcome. The model is weighted by the number of answers in each responder × exam × country group. Odds ratios (OR) are reported relative to the stated reference categories; OR < 1 means fewer correct answers than the reference group. AI – artificial-intelligence assistant; β – regression coefficient; CI – confidence interval; OR – odds ratio; SE – standard error.*

Supplementary Table S3. Portuguese PNA: AI vs physicians across knowledge domains

Predictor	β (SE)	Adjusted OR	95 % CI	*p*
Responder = AI (reference)	—	—	—	—
Physician	–1.06 (0.09)	0.35	0.29 – 0.42	<0.001
Domain = Internal medicine (reference)	—	—	—	—
Surgery	0.46 (0.09)	1.58	1.33 – 1.88	<0.001
Paediatrics	–0.13 (0.05)	0.88	0.79 – 0.98	0.025
Psychiatry	0.33 (0.07)	1.39	1.21 – 1.59	<0.001
Gynaecology/Obstetrics	0.10 (0.05)	1.11	1.00 – 1.24	0.057
Model N (answers)	41 933			

Logistic regression of Portuguese PNA answers, weighted by the number of answers in each responder × domain group. Separate 2 × 2 Fisher exact test for the paediatrics rows gave OR = 0.80, p *= 0.60. AI – artificial-intelligence assistant; β – regression coefficient; CI – confidence interval; OR – odds ratio; PNA –* Prova Nacional de Acesso*; SE – standard error.*

Supplementary Table S4. Physician specialty vs paediatrics

Specialty	Adjusted OR vs *Paediatrics (reference)*	95 % CI	*p*
Anaesthesiology	0.91	0.87 – 0.95	<0.001
Cardiology	1.62	1.54 – 1.70	<0.001
Family medicine (GP)	0.12	0.11 – 0.12	<0.001
General surgery	1.20	1.15 – 1.26	<0.001
Gynaecology/Obstetrics	2.21	2.08 – 2.35	<0.001
Internal medicine	0.60	0.58 – 0.62	<0.001
Other specialties	0.12	0.12 – 0.13	<0.001
Physicians without specialty	0.25	0.24 – 0.26	<0.001
Psychiatry	1.33	1.27 – 1.40	<0.001
Model N	221 574		

Logistic regression of physician answers, weighted by the number of answers in each specialty × country group. Paediatrics is the reference specialty. β – regression coefficient; CI – confidence interval; GP – general practitioner; OR – odds ratio; SE – standard error.

Supplementary Table S5. Sensitivity analysis: exam-level aggregation

Predictor	Adjusted OR	95 % CI	*p*
Physician vs *AI (reference)*	0.005	0.003 – 0.009	<0.001

Logistic regression with correct vs incorrect *as the outcome, weighted by the total number of answers in each responder × exam cell after collapsing each exam into a single 2 × 2 table. This coarse aggregation inflates the apparent AI advantage (reciprocal OR ≈ 198), but the direction and significance remain unchanged. AI – artificial-intelligence assistant; CI – confidence interval; OR – odds ratio.*
